# Risk aversion in the adjustment of speed-accuracy tradeoff depending on time constraints

**DOI:** 10.1038/s41598-019-48052-0

**Published:** 2019-08-13

**Authors:** Ryoji Onagawa, Masahiro Shinya, Keiji Ota, Kazutoshi Kudo

**Affiliations:** 1 Laboratory of Sports Sciences, Department of Life Sciences, Graduate School of Arts and Sciences, The University of Tokyo, Tokyo, Japan; 2 Graduate School of Integrated Arts and Sciences, Hiroshima University, Higashi-Hiroshima, Japan; 3 Institute of Engineering, Tokyo University of Agriculture and Technology, Tokyo, Japan; 4 Research Fellow of Japan Society for the Promotion of Science, Tokyo, Japan; 5 Department of Psychology, New York University, New York, United States; 6 Graduate School of Interdisciplinary Information Studies, The University of Tokyo, Tokyo, Japan

**Keywords:** Human behaviour, Reward, Decision

## Abstract

Humans are often required to make decisions under time constraints and to adjust speed-accuracy tradeoff (SAT) based on time constraints. Previous studies have investigated how humans adjust SAT depending on the time discount rate of expected gain. Although the expected gain of actions can be determined by both gain and probability, only situations where gain decreases over time have been tested. Considering the effect of risk on decision-making, the difference in time discount factors may modulate the response strategies for SAT, since temporal changes in variance of possible outcomes differ when gain or probability decreases over time. Here, we investigated the response strategies for SAT under different time discount factors. Participants were required to select one of the two options with different initial values in situations where the expected gain of options declined over time by a linear decrease in gain or probability. Comparison of response strategies between conditions revealed that response times in the gain condition were longer than those in the probability condition, possibly due to risk-aversion. These findings indicate the existence of common rules underpinning sensorimotor and economic decision-making.

## Introduction

From insects to rodents to primates, many species often face the dilemma of speed-accuracy tradeoff (SAT)^1^. SAT is evident in the relationship between movement speed and movement variability^2–4^ or between decision speed and decision accuracy^1,5–7^. For choice behavior in particular, the Hick–Hyman law^8,9^ describes the fundamental human property whereby response times for making decisions become longer as the number of options increases, since having more options requires more time to accumulate information.

When making decisions under time constraints, it is necessary to consider the competing demands of speed and accuracy depending on one’s own SAT and the given time constraints. A typical example of this would be decision-making in basketball, soccer, rugby, and American football. In many ball games, players are required to select how they move under very severe time constraints^10^, sometimes less than 1 second. Under such situations, collecting more information about a given state is beneficial but comes at the cost of sacrificing time. Therefore, if the value of allocating time to collect information is higher (e.g., defender’s pressing is loose), taking a longer time to make a decision may produce better outcomes than rushing to make a decision. In contrast, if the value of allocating time is less (e.g., defender’s pressing is severe), making a decision more rapidly may lead to better outcomes. Therefore, optimizing time allocation is indispensable for maximizing performance.

Previous studies have investigated how SAT is adjusted under situations in which the gains of options gradually decreased over time^7,11^. Theoretically, the expected gain of an option is determined by both gain and probability of success; thus, situations where probability changes over time should also be considered. For instance, in basketball, it has been reported that the shot success rate was decreased with an increase in the time required to take the shot^12^. Similarly, in several sports, the expected gain in possible action is affected by probability. It remains unclear how people adjust SAT depending on time constraints when the success probability of options decreases with increasing time spent on making decisions. Further, it remains unknown whether the adjustment of SAT according to a given time constraint varies between situations in which either gain or probability decrease over time.

Humans frequently show bias regarding the optimal choice to maximize expected rewards due to their attitudes towards outcome variance (i.e., risk) in economic and motor decision tasks^13–18^. Most individuals prefer certainty in economic decisions^13,14^ but tend to take risks in motor decisions^15–18^. Thus, both the expected gain and variance of possible outcomes are significant factors that influence decision-making.

In this study, time changes in the variance of possible outcomes differed depending on whether the time discount factor was gain or probability. Therefore, even though the time change of the expected gain was equal between time-discounted factors, the difference in variability of possible outcomes may have promoted different strategies depending on risk preference. Here, we investigated how humans adopted their own SAT according to differences in time-discount factors.

**Figure 1:**
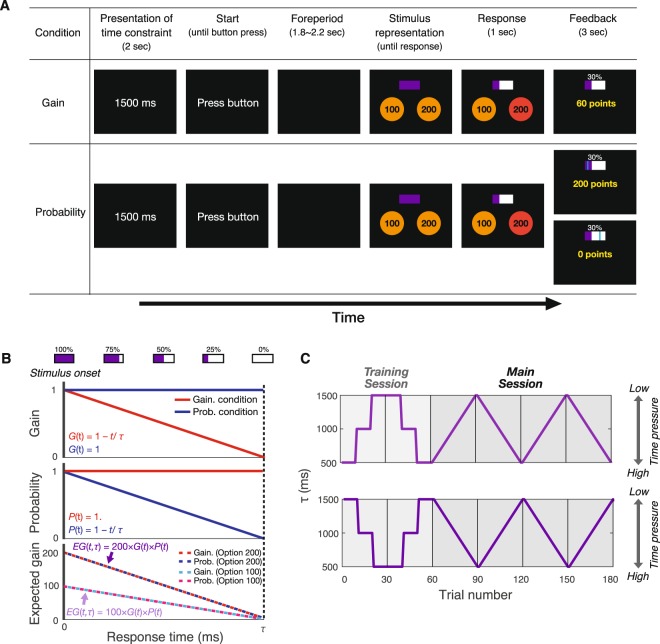
Experimental setup. (**A**) Task sequences of the gain and probability-decrease conditions. At the beginning of the task, time interval until gain/probability decreased to 0 (τ ms) was presented at the center of the screen. The participants initialized a trial by pressing a button. After a random fore-period, two choice stimuli and a time bar (the purple bar) were presented. The number presented on the stimuli showed the initial values (100 or 200). Expected gain of each option decreased linearly over time from the initial values to zero points by decreasing gain or probability. The time bar showed the gain/probability at that time. Participants pressed one of two buttons corresponding to the two choice stimuli at any time. The color of the selected stimuli turned to red, after which time-discounted score [e.g., 60 points (200 points × 0.3) in panel A] and gain (e.g., 30% in panel A) were presented as feedback in the gain-decrease condition. Score (e.g., 0 or 200 points in panel A), probability (e.g., 30% in panel A), and a blue line within the range of the time bar were presented in the probability-decrease condition. The position of the blue line was determined by a uniform random number. Participants obtained scores only when this line fell within the purple area. (**B**) Gain/probability/expected gain function. The upper, middle, and lower panels show gain function, probability function, and expected gain function of each option in both conditions, respectively. When τ was the same value, the expected gain depending on time was equivalent between two conditions. (**C**) Trial sequences of *τ*. We used two sequences of τ and changed the sequence between conditions. In the training session, participants performed 20 trials under three levels of time pressure (τ = 500, 1000, 1500 ms). In the main session, τ ascended or descended across trials from 500 to 1500 ms [step size of τ change was approximately 34.5 (~1000/29) ms]. The order of trial sequences of τ and condition (gain or probability) were counterbalanced among participants.

In our task (Fig. 1A), participants (*N* = 12) were required to select one of two choice stimuli presented on the left- or right-side on a screen. These stimuli (Option 100 or Option 200) had different initial values (100 or 200 points) at the presentation onset of the choice stimuli. The position of each stimulus was randomized across trials, so that the participants could not predict locations of either Option 100 or Option 200. The expected gain of the options gradually decreased over time and became 0 after a time interval (τ) from the stimulus onset (Fig. 1B). We manipulated either gain or probability to discount the options’ expected gain (Fig. 1B). In the gain-decrease conditions, the values were discounted as time passed. In the probability-decrease condition, the probability that a chosen option indicated success decreased over time. Gain and probability had the same influence on the expected gain of each option. The participants chose one of two options by pressing a button on a manipulation pad. We set the time interval (τ) until gain/probability became 0 between the period from 500 to 1500 ms (Fig. 1C). The participants were instructed to maximize the average score in each experimental block.

In this task, since the participants could not predict location of each option, choosing the larger initial value option (i.e., Option 200) resulted in required more time than did choosing either option as early as possible without searching options, in accordance with the Hick–Hyman law^8,9^. The former and latter response strategies correspond to choice and simple reactions, respectively. If there were no time constraints (i.e., the expected gain did not decrease over time), choice reaction was the best solution as a matter of course. However, when the expected gain was discounted over time, taking more time to make a decision would lead to a less expected outcome. If the time interval until gain/probability became 0 was extremely short, selecting either option by guesswork would lead to a higher expected outcome.

We had three hypotheses. If the participants were not sensitive to the variance (risk) of possible outcomes and only considered the expected outcomes (i.e., risk-neutral), the response time would be the same between two conditions because the time changes in expected outcomes were controlled to be equal. If the participants avoided variance in possible outcomes and aimed for less variable outcomes (i.e., risk-averse), response times would shift to reduce the variance (i.e., a longer response time would be observed in the gain-decrease condition and a shorter response time would be observed in the probability-decrease condition). If the participants sought variance in possible outcomes and aimed for a highly variable outcome (i.e., risk-seeking), response times would also shift to increase the variance (i.e., a shorter response time would be observed in the gain-decrease condition and a longer response time would be observed in the probability-decrease condition). We investigated whether response strategies under time constraints differed between the gain-decrease and probability-decrease conditions.

## Results

**Figure 2:**
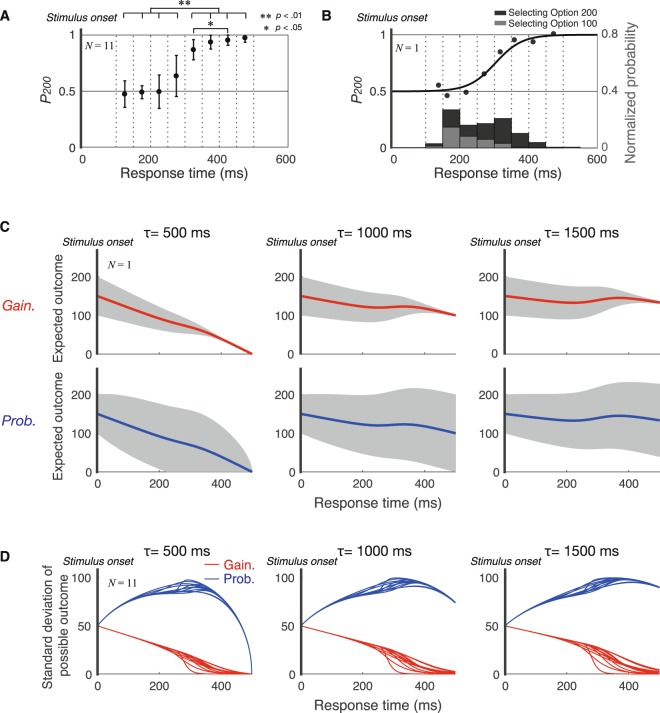
Three steps to estimate time changes in variance of possible outcomes. (**A**) Relationship between response time and probability of selecting Option 200. The black circles indicate the mean of the probability of each participant selecting Option 200 (*P*_200_) and the black bars indicate between-participant SD of *P*_200_. The asterisks above a line indicates that the difference between *P*_200_ for the corresponding response times was significant (paired *t*-test with Bonferroni correction). These results indicated that the speed-accuracy tradeoff was confirmed for this task. (**B**) Histogram of response times for selecting Option 100 (gray) and Option 200 (black). The probability of selecting Option 200 (*P*_200_) is plotted as a function of the response time binned in eight equal intervals (50 ms) from 100 to 500 ms. The response time include both the gain-decrease and probability-decrease condition. The black curve shows the model fit using modified logistic regression and indicates the relationship between the response time and *P*_200_ (i.e., participant’s own speed-accuracy tradeoff). The histogram shows the distribution of the response time across equal intervals (50 ms). The figures obtained from each participant are shown in Supplementary Fig. 1. (**C**) Time changes in the expected outcomes in the gain-decrease (red) and probability-decrease (blue) conditions. The shaded gray area indicates the variance of possible outcomes. These were based on data from an individual participant. The expected outcomes (*EO*) depending on time were estimated using participants’ speed-accuracy tradeoff (**A**). Since the expected gain of each option at each time point was equivalent between both conditions, the expected outcome depending on time was the same between conditions. (**D**) Time changes in variance of possible outcomes. The blue and red lines show the time changes in variance (risk) of possible outcomes for each participant in gain-decrease and probability-decrease conditions, respectively. In the gain-decrease condition, when participants selected Option 200, variance became small. In the probability-decrease condition, variance became large when participants selected Option 200, since the probability of obtaining no reward or obtaining 200 points simultaneously increased.

We first estimated how the expected outcomes and variance (i.e., risk) of possible outcomes changed according to response time. For this, we estimated participants’ SAT: the relationship between response time and probability of selecting the larger initial value option (Option 200) *P*_200_. We calculated response frequency for selecting Option 100 and Option 200 (gray and black bars in Fig. 2B) binned in eight equal intervals (50 ms) within 100 to 500 ms and then calculated the probability of choosing Option 200 in each bin (black circles in Fig. 2B). One-way repeated measures ANOVA for *P*_200_, using response time as an independent variable, revealed that there were significant main effects (*F*[7, 70]* = *58.148, ${{\eta }_{p}}^{2}$ = 0.853, *p* < 0.001). Post-hoc paired *t*-tests with Bonferroni correction revealed that there were significant differences between four earlier response time (100–150, 150–200, 200–250, and 250–300 ms) and four later response time (300–350, 350–400, 400–450, and 450–500 ms) (*ps* < 0.01, ** in Fig. 2A). These results indicated that the participants selected Option 200 more frequently as their response times increased (Fig. 2A) (individual data in Supplementary Fig. 1). We next estimated participants’ own SAT (black line in Fig. 2B) by fitting a generalized linear model with modified Probit link function^19^ to the response probability data (black circles).

Using the fitted curve between *P*_200_ and response times, we estimated the time change of expected outcome in both the gain-decrease condition and probability-decrease condition (Fig. 2C) (individual data in Supplementary Fig. 2). The expected outcome corresponding to response time was determined by expected gain which decreased over time and *P*_200_ which increased over time. There were similar time-change patterns of expected outcomes among the participants (Supplementary Fig. 1). Because the time discount of gain and probability was the same (Fig. 1B), the time change of expected outcomes was also the same between both conditions (difference between red and blue lines in Fig. 2C).

Next, we estimated time changes in the variance of possible outcomes; namely, how possible outcomes varied from expected outcomes (Fig. 2D). In the gain-decrease condition, the variance of possible outcomes decreased over time because participants could search for time-discounted Option 200 by increasing time spent. In the probability-decrease condition, *P*_200_ increased whereas the probability of the selected option being successful decreased over time. Therefore, the variance of possible outcomes increased and subsequently reached 0. The features of temporal-change patterns in the variance of possible outcomes for each condition were robust for all participants (Fig. 2D) (individual data in Supplementary Fig. 3). If participants avoided variance of possible outcomes (i.e., risk-averse behavior), they would respond slowly in the gain-decrease condition and would respond quickly in the probability-decrease condition because this strategy would reduce the variance of possible outcomes.

**Figure 3:**
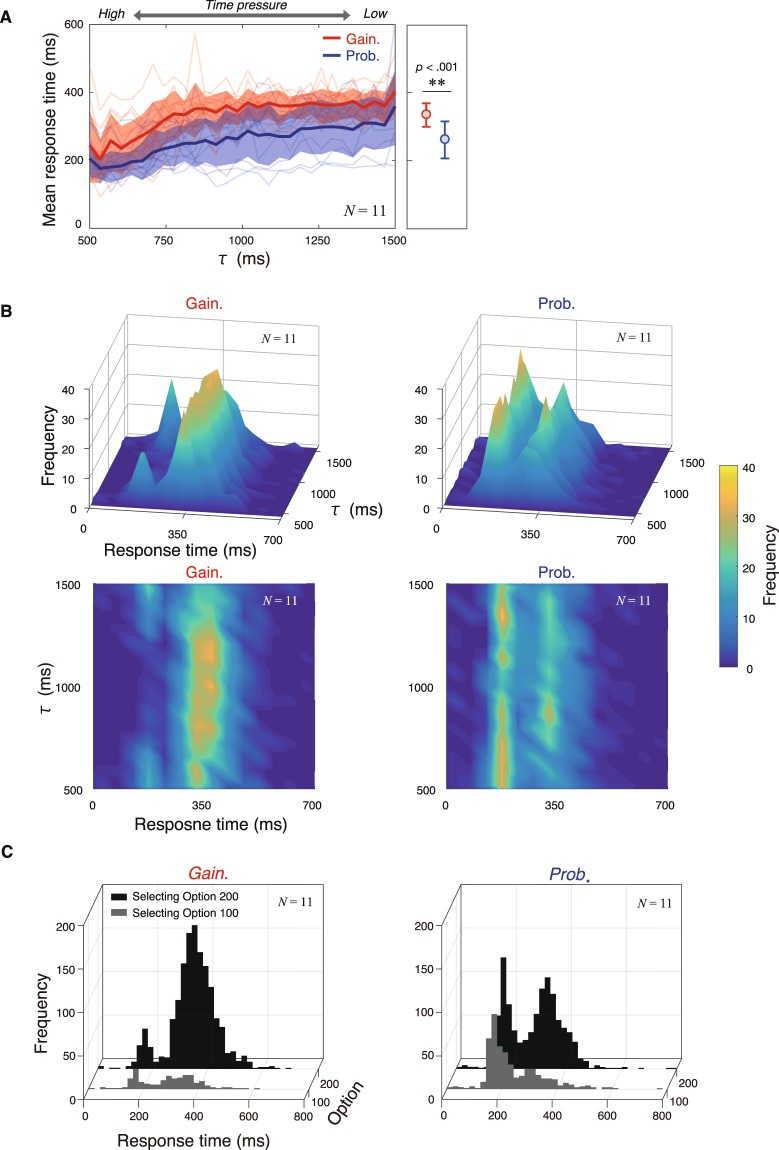
Different response strategy patterns between the gain-decrease and probability-decrease conditions. (**A**) Comparison of mean response times between gain-decrease and probability-decrease condition. The red and blue thick solid lines indicate the mean of the mean response times of each participant corresponding to time constraint τ in the gain-decrease and probability-decrease conditions, respectively. The shaded areas indicate between-participant SD of the mean response times. The thin lines indicate the mean response times for each participant. There was a significant difference between the gain and probability-decrease conditions, and between the mean response times among time constraints. Participants adjusted speed-accuracy tradeoff corresponding to time constraints and task conditions. (**B**) Histogram of response times corresponding to time constraints. The left and right panels included the data for all participants in the gain and probability-decrease condition, respectively. The upper panels is viewed from (azimuth, elevation) = (10, 20), and the lower panels is viewed from (azimuth, elevation) = (0, 90), where, the azimuth is a polar angle in the x (RT)-y (τ) plane with positive angles indicating counterclockwise rotation of the viewpoint from -y axis, and the elevation is an angle above the x-y plane. (**C**) Histogram of response time pooled among time constraints. Data for all participants were included. Black and gray histograms indicate the trials in which participants selected Option 200 and Option 100, respectively.

Figure 3A illustrates the mean response time corresponding to the time interval until gain/probability became 0 in both the gain-decrease and probability-decrease conditions. Paired *t*-test for mean-response time (including all responses in the main session) between conditions revealed that there was a significant difference between conditions (*t*[10] = 5.0735, *dz* = 1.53, *p* < 0.001). Two-way repeated measures ANOVA using task condition and time interval until gain/probability became 0 as independent variables revealed significant main effects of task condition (*F*[1, 29] = 27.206, ${{\eta }_{p}}^{2}$ = 0.73, *p* < 0.001) and time interval until gain/probability became 0 (*F*[29, 290] = 16.461,$\,{{\eta }_{p}}^{2}$ = 0.62, *p* < 0.001). There was no significant interaction (*F*[29, 290] = 1.205,$\,{{\eta }_{p}}^{2}$ = 0.11, *p* > 0.05). These results suggested that response times became shorter under severe time constraints, and that response time in the gain-decrease condition was longer than that in the probability-decrease condition.

Figure 3B,C illustrate the histogram of response times in the gain-decrease and probability-decrease conditions, respectively. Although both histograms have bimodal peaks, a faster peak appeared more frequently in the probability-decrease condition. Because the response histogram did not differ among time intervals until gain/probability became 0 (Fig. 3B), we pooled the response time data among time constraints (Fig. 3C). Similarly, the response time distribution had a bimodal property, indicating that participants used discrete response patterns (earlier response patterns appeared as a simple reaction, and later response patterns appeared as a choice reaction). The bimodal property in the response time distribution can be explained by the phase transition model^20^ assuming that two responses transfer between guess mode (simple reaction) and stimulus-controlled mode (choice reaction).

**Figure 4:**
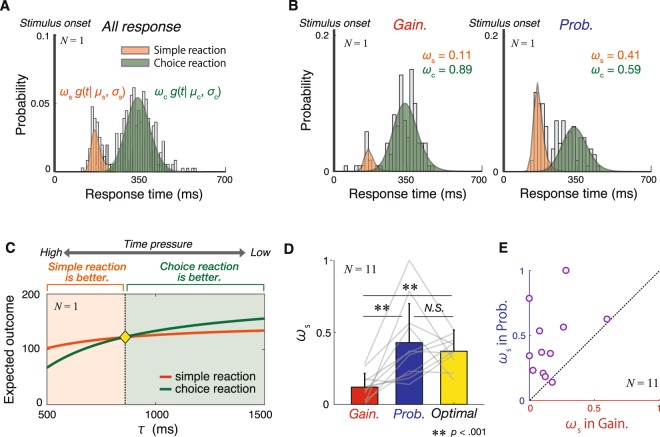
Evaluation of the optimality of response-strategy selection. (**A**) Gaussian mixture model (GMM) fitting. The gray histogram indicates the response time distribution of an individual participant (including all response data). The orange and green shaded areas show two components of Gaussian distribution estimated by GMM. The orange and green components indicate a simple reaction pattern and choice reaction pattern, respectively. The figures for each participant are shown in Supplementary Fig. 5. (**B**) GMM for each condition. The upper and lower panels show response time distribution in the gain and probability-decrease conditions, respectively (based on data from the same participant depicted in Fig. 4B). The orange and green shaded areas indicate the components of Gaussian distributions determined by the ratio of simple reaction pattern in each condition (${\varpi }_{s-gain}$ or ${\varpi }_{s-prob}$) which was fitted to observed data. The figures for each participant are shown in Supplementary Fig. 6. (**C**) Estimation of optimal weighting of the simple reaction ${\varpi }_{s}$. The orange and green lines indicate the expected gain of simple and choice reaction patterns corresponding to τ, respectively. The yellow diamond shows the point of indifference between response patterns, indicating the optimal switching point. The figures for each participant are shown in Supplementary Fig. 7. (**D**) Comparison of weighting of the simple reaction. The bars show mean ${\varpi }_{s}$ in each condition and mean optimal ${\varpi }_{s}$ among participants. The thin gray lines indicate individual data. There were significant differences in the weighting of simple reaction patterns in the gain-decrease condition ${\varpi }_{s\_gain}$ compared to that in the probability-decrease condition. (**E**) Comparison between weighting of the simple reaction between conditions. The purple circles indicate individual data. The bias of response-strategy selection was highly consistent among participants.

To further investigate how participants weighted their responses for simple and choice reactions in both conditions, we estimated the mean reaction time and standard deviation corresponding to simple and choice reactions as the ability of each reaction pattern for each participant using Gaussian mixture model (GMM) fitting. Our model assumption was that participants had the same ability for performing simple and choice reactions in both conditions since sensory input and motor output were the same between conditions (see Methods). We fitted GMM to the response time data including both the gain and probability-decrease conditions in each participant (Fig. 4A). GMM with two distributions has six free parameters (${\varpi }_{s},\,{\varpi }_{c},\,{\mu }_{s},\,{\mu }_{c},\,{\sigma }_{s},\,{\sigma }_{c}$), where $\varpi$ is a weighting parameter, *μ* is the mean of Gaussian distribution, and *σ* is the standard deviation of Gaussian distribution. The subscript *s* indicates a simple reaction for each parameter, and the subscript *c* indicates a choice reaction for each parameter. The fitted parameters for each participant are described in Supplementary Table 1. The estimated mean and standard deviation in the simple and choice reactions (${\mu }_{s},\,{\mu }_{c},\,{\sigma }_{s},\,{\sigma }_{c}$) were considered to reflect reaction ability in SAT. For given these four parameters, we again fit GMM which had two free parameters (${\varpi }_{s},\,{\varpi }_{c}$) to the data in the gain and probability-decrease condition separately. Therefore, we obtained weighting parameters for the simple reaction (${\varpi }_{s\_gain}$, ${\varpi }_{s\_prob}$) and choice reaction ($1-{\varpi }_{s\_gain}$, $1-{\varpi }_{s\_prob}$) in the gain and probability-decrease conditions, respectively (Fig. 4B). The weighting of simple reaction in the gain-decrease condition was less than that in the probability-decrease condition (Fig. 4B,D), indicating that participants adopted the simple reaction less frequently in the gain-decrease condition.

To assess whether participants used the simple reaction optimally, we estimated the optimal ratio for using the simple reaction (${\varpi }_{s\_opt}$) based on Bayesian decision theory^21–23^. To estimate the expected outcomes for each reaction pattern, we used three factors: the estimated parameters reflecting the ability of the simple and choice reaction patterns (*μ*_*s*_, *μ*_*c*_, *σ*_*s*_, *σ*_*c*_), the probability of selecting the larger initial value in each reaction pattern (if participants used the simple reaction, the probability of selecting Option 200 would indicate a 50% chance, and if participants used the choice reaction, the probability of selecting Option 200 would be approximately 100%), and the expected outcome corresponding to response times according to time intervals until gain/probability became 0. We then calculated the expected outcomes for both the simple and choice reaction patterns for a given time interval (Fig. 4C). When time constraints were severe, the expected outcomes for the simple reaction pattern was higher than that for the choice reaction pattern. In contrast, when time constraints were not severe, the expected outcomes for the choice reaction pattern were higher. We defined a time constant in which the expected outcomes were equal for both reaction patterns (yellow diamond in Fig. 4C). When the time interval for given a trial was less than this time constraint, using the simple reaction pattern was better to maximize expected outcomes. We defined the optimal weighting ratio for using the simple reaction pattern ${\varpi }_{s\_opt}$ as the ratio of expected outcomes for the simple reaction pattern being better than that for the choice reaction pattern between *τ* = 500 ms and *τ* = 1500 (red region in Fig. 4C).

Friedman test was performed to compare the estimated weighting of simple reaction in the gain-decrease condition ${\varpi }_{s\_gain}$, probability-decrease condition ${\varpi }_{s\_prob}$, and optimal weighting ratio ${\varpi }_{s\_opt}$. We observed a significant main effect of condition (*χ*^2^[2 ]= 13.63, *W* = 0.62, *p* < 0.001; Fig. 4D). Post-hoc Wilcoxon signed-rank test revealed that the weighting of simple reaction in the gain-decrease condition was significantly lower than that in the probability-decrease condition (*Z* = −2.85, *p* < 0.05, Bonferroni correction) and the optimal weighting ratio (*Z* = −2.93, *p* < 0.05, Bonferroni correction). There was no significant difference between the weighting of simple reaction in the probability-decrease condition and optimal weighting ratio (*Z* = −0.44, *p* > 0.05, Bonferroni correction). Additionally, the direction of bias was highly consistent among participants (Fig. 4E). These results indicate that participants less frequently used the simple reaction compared to the optimal ratio when gain decreased over time.

## Discussion

Previous studies have shown that humans adaptively change their movements and decision speed when the gain of options gradually decreases with time^7,11^. We focused on the differences in time-discount factors between gain and probability and investigated the use of response strategies under different time-discount factors. We hypothesized that the participants changed their response strategies between conditions taking into account risk-dependent human decision-making, since the variance of possible outcomes changed between conditions (Fig. 2C,D). Indeed, we observed that participants more frequently used the choice reaction pattern in the gain-decrease condition than in the probability-decrease condition (Fig. 3B,C), which resulted in shorter reaction times in the probability-decrease condition (Fig. 3A). Longer reaction times reduced the variance of possible outcomes in the gain-decrease condition whereas shorter reaction times reduced the variance in the probability-decrease condition (Fig. 3C). Therefore, reaction time was biased toward risk-aversion. Furthermore, participants’ frequency of simple reaction patterns in the gain-decrease condition was less than the optimal frequency for maximizing expected outcomes (Fig. 4D).

In our study, participants were required to select either smaller (100) or larger (200) initial value options under time constraints. They could freely decide when to pick an option under the gain/probability of the option decreasing as time passed (Fig. 1). Although they could use any strategy for how much time they spent from stimulus onset, we observed that response times were bimodally distributed (Fig. 3C). This property indicated that participants adopted two discrete response strategies, and they seemed to adapt to the degree of time pressure by switching between two response patterns. Under the framework of the Hick–Hyman theory^8,9^, response time increases as the number of stimulus–response alternatives increases. Thus, the earlier and later response patterns corresponded to the simple and choice reactions, respectively. These results indicated that response-strategy selection can be interpreted as an issue of selection between two response patterns (simple or choice reaction), rather than the selection between continuous and infinite response strategies. Notably, the simple reaction was to select either Option 100 or Option 200 as soon as possible after the trial started, leading to a low time discount rate but approximately 50:50 chance for selecting Option 200. The choice reaction was to select Option 200 after by sacrificing time-discounting, leading to a high time discount and high probability of selecting Option 200 (approximately 100% chance).

Since the expected outcomes were equivalent between conditions (Fig. 2C), the weighting ratio should have been equal between conditions if participants took only the expected outcomes into account. However, the weighting ratio of the simple reaction in the gain-decrease condition was less than that in the probability-decrease condition (Fig. 4D). These results indicated that participants considered the variance of possible outcomes, and this distortion reflected their risk-aversion. This tendency is inconsistent with previous studies reporting that humans tend to be risk-seeking in sensorimotor tasks^15–18^. These studies only manipulated gain to control the expected gain. However, by comparing the conditions manipulating probability or gain, we confirmed risk-averse tendency in sensorimotor decision tasks.

Markowitz’s risk-return model^24^, known as Modern portfolio theory, suggests that the value *U*(*x*) of an investment x is modeled as a trade-off between the expected outcomes (mean return) *E*(*x*) and the variability of the outcomes (risk) *Var*(*x*), such that $U(x)=E(x)-\theta Var(x)$^25^. *θ* expresses the decision maker’s risk attitude: risk-neutral decision-makers are only sensitive to the expected outcomes (i.e., *θ* = 0) whereas risk-averse individuals discount outcome variability (i.e., *θ* > 0), and risk-seekers consider it a bonus (i.e., *θ* < 0). In this regard, the patterned deviation of response strategies suggests that participants in this study discounted the variance of possible outcomes (i.e., *θ* > 0) since the shift of response strategy between conditions was in the direction of variance reduction.

Different views of human decision-making include prospect theory^13^ and cumulative prospect theory^26^. These theories quantify risk-seeking or risk-averse behavior through distortions in the value function and probability weighting function^18^. In these theories, outcomes values depend on a reference point^27^, and humans exhibit higher sensitivity to loss than to gain, relative to their reference point. If participants avoided losses in the probability-decrease condition, they would prefer the simple reaction because the choice reaction increased the probability of rewarding no points. If they also avoided losses in the gain-decrease condition, they would prefer the choice reaction because the simple reaction increased the probability of lower outcomes by selecting Option 100. Therefore, the valuation of possible plans was considered to obey common rules to those of loss-aversion in economic decisions.

In the gain-decrease condition, the weighting of the simple reaction was significantly smaller than the optimal weighting, indicating that participants less frequently used the simple reaction. In contrast, the weighting ratio for the simple reaction in the probability-decrease condition was not significantly different from the optimal ratio. These results suggest that humans sub-optimally over-searched for a higher initial value option (i.e., Option 200) in the gain-decrease condition.

One of the difficulties when investigating sensorimotor decision-making is that the estimation of one’s own ability (for example, SAT in the current study) is involved in the evaluation of possible plans, which differs from economic decision-making. The perception of the sensory consequences of one’s actions is more biased toward success relative to the perception of observed actions^28^, and motor variance represented by an agent is underestimated relative to actual variance^29,30^. Therefore, it is possible that misestimating one’s own SAT led to deviation from the optimal selection of response strategy. However, because the same sensory input and motor output were required in both conditions, participants would have equally misestimated their own SAT in each condition. Therefore, misestimation of SAT is unlikely to fully explain the observed differences in response strategies.

The experimental setting of the two equivalent conditions allowed us to directly examine the effects of distorted utility function on sensorimotor decision-making because the equivalent setting excluded the effects of distorted probability estimation in one’s own ability of SAT. Therefore, when investigating decision-making processes in sensorimotor control, manipulating gain and/or probability would be an effective way to control the expected gain of options.

This study investigated how time discount factors (gain and probability) that determined the expected gain of options affected the selection of response strategies. We observed that participants took longer to respond when the gain decreased over time compared to when the probability decreased. Participants frequently adopted the choice reaction strategy in the gain-decrease condition, whereas they adopted either the simple or choice reaction in the probability-decrease condition. This strategy shift could be interpreted as risk aversion, which is inconsistent with the evidence of risk-seeking behavior reported in many studies of sensorimotor decision-making which only manipulated the gain factor. Therefore, we suggest adding the probability factor in future studies to investigate human decision-making strategies in motor tasks.

## Methods

### Participants

Twelve healthy right-handed adults (nine males, three females; mean age: 24.3 ± 1.8 years) were recruited. The participants were unaware of the purpose of the experiment. This study was approved by the Ethics Committee of the Graduate School of Arts and Sciences, the University of Tokyo. The approved guidelines were adhered to for all experimental procedures. Informed consent was provided by each participant before the experiments in written format.

### Experimental task and procedures

Participants sat in a quiet, dim room. Their head was positioned on a chin rest with an adjustable forehead rest 45 cm in front of a monitor (ASUS, VG248QE, 24 inches, 1920 × 1080 pixels, vertical refresh rate 100 Hz) that was used to present stimuli. The participants held a manipulation pad (Microsoft, 7MN-00005). All stimuli were controlled using Psychophysics toolbox^31–33^ in MATLAB.

For each gain-decrease and probability-decrease condition, there were two sessions: *training* and *main*. The training session consisted of two sets of 30 trials. The main session consisted of four sets of 30 trials. In each set of the training session, participants performed the task under high time pressure (τ = 500 ms), moderate time pressure (τ = 1000 ms), and low time pressure (τ = 1500 ms) for 10 trials each. In each set of the main session, τ initialized at 500 ms and gradually increased, or initialized at 1500 ms and gradually decreased. Two sequences of τ were prepared (Fig. 1B). Participants performed the training and main sessions consecutively in each condition. Participants performed 360 trials (180 trials [training session: 60, main session: 120] × 2 conditions [gain-decrease and probability-decrease condition]) in total. The order of conditions and sequence of τ were counterbalanced across participants.

Participants chose either one of two options which had different initial values (100 or 200 points) by manually pressing a button corresponding with the two options (Fig. 1A). The expected gain of each option decreased linearly from the initial values to zero points over time by decreasing gain or probability (Fig. 1C) in accordance with previous protocols^7,11^. In both conditions, the time interval until gain/probability became 0 τ was represented at the center of the monitor at the beginning of the task (Fig. 1A). Participants were instructed to maximize the average score in each set.

### Gain-decrease condition

After the presentation of the time interval for 2 sec, participants initialized a trial by pressing a button. After the random fore-period interval (1800–2200 ms), two circles representing initial values (100 or 200 points) and a purple bar were presented on the screen. As time passed, the size of the purple bar (the time bar) decreased continuously. Participants were informed that the resting purple bar indicated the gain (i.e., rate of acquired score). When the purple bar reached the leftmost point, the gain became 0. Participants pressed either one of two buttons corresponding with two options when they decided on the option. After their choice, the gain at that moment (for example, 30% in Fig. 1A) and time discounted score (for example, 60 points [200 points × 0.3] in Fig. 1A) were presented for 3 sec as performance feedback.

The gain function *G*(*t*, *τ*) and probability function *P*(*t*, *τ*) in the gain-decrease condition were determined by the following equations

1 $$G(t,\tau )=\{\begin{array}{ll}1-\frac{t}{\tau }, & {\rm{if}}\,t\le \tau \\ 0, & \,{\rm{if}}\,t > \tau \end{array}$$

2 $$P(t,\tau )=\{\begin{array}{ll}1, & {\rm{if}}\,t\le \tau \\ 0, & {\rm{if}}\,t > \tau \end{array}$$

where *t* was the response time when participants pressed a button, and *τ* was the time interval until gain/probability became 0.

### Probability-decrease condition

The trial sequence in the probability-decrease condition was the same as that in the gain-decrease condition except for the feedback. In the probability-decrease condition, the resting purple bar indicated how much probability remained. Participants could obtain the initial value (100 or 200 points) with the probability according to response time. After they selected an option, the score (0, 100, or 200 points) and probability (for example, 30% in Fig. 1A) were presented as performance feedback. A blue line was shown within the bar to determine the score. If the blue bar fell within the purple bar, participants earned the selected initial score (200 points in Fig. 1A). If the blue bar fell within the white bar, the score was 0 (Fig. 1A). The position of the blue line was unpredictable and determined randomly.

The gain function *G*(*t*, *τ*) and probability function *P*(*t*, *τ*) in the probability-decrease condition were determined by the following equations

3 $$G(t,\tau )=\{\begin{array}{ll}1, & {\rm{if}}\,t\le \tau \\ 0, & {\rm{if}}\,t > \tau \end{array}$$

4 $$P(t,\tau )=\{\begin{array}{ll}1-\frac{t}{\tau }, & {\rm{if}}\,t\le \tau \\ 0, & {\rm{if}}\,t > \tau \end{array}$$

where *t* was elapsed time from stimulus onset, and *τ* was the time that probability became zero from stimulus onset.

### Data analysis

In each trial, we recorded the response time (*button press time* − *onset of stimulus*), the selected initial value, score, and value of gain/probability. The data were collected at a sampling rate of 100 Hz. One participant adopted the same response strategy for all time intervals until gain/probability became 0. We excluded the data for this participant from the analysis because the selection of response strategy (simple or choice) could not be distinguished.

### Model assumptions

#### Estimation of time changes in variance of possible outcomes

To estimate the time-dependent variance (i.e., risk) of possible outcomes, we performed three processes. First, we estimated participants’ speed-accuracy tradeoff (SAT) between the probability of choosing a larger initial value option *P*_200_ and the response time. The mean response time binned in eight equal intervals within 100 to 500 ms and the corresponding *P*_200_ were calculated for each participant. We ran a maximum likelihood estimation to the relationship between *P*_200_ and reaction time using a generalized linear model with a modified Probit link function. The modified Probit link function can be used to estimate a rescaled lower asymptote *C* and an upper asymptote *D* in a two alternative forced choice task. In an ideal two alternatives forced choice task, the percentage correct varies from 50% to 100% such that *C* = 0.5 and *D* = 1.0. *C* and *D* are incorporated into probit analysis by assuming the cumulative normal function (*F*) in which the probability changes from 0 to 1.0. Using Abbott’s formula to obtain a percentage selecting Option 200 (*P*_200_), whose limits are C and D:

5 $${P}_{200}(t)=C+(D-C)F(t|{\mu }_{p},{\sigma }_{p})$$

where t is time. $F(t|{\mu }_{p},{\sigma }_{p})$ is the cumulative normal function. *μ*_*p*_ is the mean and *σ*_*p*_ is the standard deviation for the cumulative normal function. *P*_200_(*t*) satisfies the constraint that ${P}_{200}(t)=1-{P}_{100}(t)$. Next, we calculated time changes of the expected outcomes. Using “glmfit” function in Matlab, we estimated the two free parameters (*μ*_*p*_, *σ*_*p*_) describing the relationship between *P*_200_ and reaction time.

Second, we estimated the expected outcome depending on time *EO*(*t*, *τ*) using SAT as below:

6 $$EO(t,\tau )=(1-{P}_{200}(t))\times 100\times G(t,\tau )\times P(t,\tau )+{P}_{200}(t)\times 200\times G(t,\tau )\times P(t,\tau ),$$

where *G*(*t*, *τ*) is the gain function and *P*(*t*, *τ*) is the probability function. The first and second terms are the expected outcomes for Option 100 and Option 200, respectively. The expected outcome function is illustrated in Fig. 2C (thick lines). Of note, the expected outcome depending on time *EO*(*t*, *τ*) was the same between the gain and probability-decrease conditions. Finally, we calculated the variance of possible outcomes depending on time *V*(*t*, *τ*) in two conditions as follows:

7 $$\begin{array}{c}\{\begin{array}{rcl}{V}_{gain}(t,\tau ) & = & \begin{array}{c}{P}_{200}(t)\,\times {(EO(t,\tau )-200\times Gain(t,\tau ))}^{2}\\ +\,(1-{P}_{200}(t))\times {(EG(t,\tau )-100\times Gain(t,\tau ))}^{2}\end{array}\\ {V}_{prob}(t,\tau ) & = & \begin{array}{c}{P}_{200}(t)\times ({(EO(t,\tau )-200)}^{2}\times P(t,\tau )+(1-P(t,\tau ))\times {(EO(t,\tau )-0)}^{2})\,\\ \,+\,(1-{P}_{200}(t))\times (P(t,\tau )\times {(EO(t,\tau )-100)}^{2}+(1-P(t,\tau ))\times {(EO(t,\tau )-0)}^{2})\end{array}\end{array}\end{array}$$

The standard deviation of the possible outcomes in each condition is illustrated in Fig. 2D.

#### Gaussian mixture model fitting to the response time distribution

We used a Gaussian mixture model (GMM)^34^ to distinguish participants’ response patterns. We generated a histogram of the response times pooled for two conditions (Fig. 4A). The histogram revealed a bimodal distribution for response times, indicating that participants adopted two response patterns: simple and choice reactions.

A GMM is a parametric probability density function representing the weighted sum of Gaussian distributions. Because the response time distribution was bimodal, we fitted the response time data to two components of Gaussian distributions as follows:

8 $$p(t|{\varpi }_{s},\,{\varpi }_{c},\,{\mu }_{s},\,{\mu }_{c},\,{\sigma }_{s},\,{\sigma }_{c})={\varpi }_{s}\,g(t|{\mu }_{s},{\sigma }_{s})+{\varpi }_{c}\,g(t|{\mu }_{c},{\sigma }_{c}),$$

where *t* is time, ${\varpi }_{s}$ and ${\varpi }_{c}$ are weighting parameters for simple reaction and choice reaction pattern, respectively. $g(t|{\mu }_{s},{\sigma }_{s})$ and $g(t|{\mu }_{c},{\sigma }_{c})$ are the components of Gaussian distribution with mean (*μ*_*s*_, *μ*_*c*_) and standard deviation (*σ*_*s*_, *σ*_*c*_) in simple reaction and choice reaction, respectively. The sum of two weights satisfies the constraint that ${\varpi }_{s}+{\varpi }_{c}=1$. We estimated six free parameters (${\varpi }_{s}$, ${\varpi }_{c}$, *μ*_*s*_, *μ*_*c*_, *σ*_*s*_, *σ*_*c*_) for each participant’s response time distribution using *fitgmdist* function in Matlab.

#### Estimating the ratio of simple reaction

To compare response patterns between conditions, the parameters (*μ*_*s*_, *μ*_*c*_, *σ*_*s*_, *σ*_*c*_) estimated in GMM fitting were fixed as the parameters showing participant’s own performance of simple and choice reaction patterns and used to estimate the weighting of simple reaction patterns in each condition (${\varpi }_{s\_gain}$, ${\varpi }_{s\_prob}$). We estimated ${\varpi }_{s\_gain}$ and ${\varpi }_{s\_prob}$ which best captured the observed response time distribution in each condition using maximum likelihood estimation (*mle* function in Matlab).

#### Estimating expected outcomes of each response pattern

To evaluate the optimality of the two reaction patterns corresponding to the level of time constraints, we calculated the expected outcomes of each reaction pattern. Expected outcomes of each reaction pattern are as follows:

9 $$\{\begin{array}{rcl}E{O}_{s}(\tau ) & = & {\int }_{0}^{\tau }G(t,\tau )\times P(t,\tau )\times g(t|{\mu }_{s},{\sigma }_{s})dt\\ E{O}_{c}(\tau ) & = & {\int }_{0}^{\tau }G(t,\tau )\times P(t,\tau )\times g(t|{\mu }_{c},{\sigma }_{c})dt\end{array}$$

We then estimated τ^*^, that is, $E{O}_{s}(\tau )-E{O}_{c}(\tau )=0$ as the optimal switching point (yellow diamond in Fig. 4C). If a given time interval until gain/probability became 0 is shorter than τ^*^, the simple reaction becomes a better response strategy. If a given time interval is longer than τ^*^, choice reaction becomes better.

### Statistical analysis

We conducted a one-way (8 [response times binned in eight equal intervals within 100 to 500 ms]) repeated-measures ANOVA on *P*_200_ as an independent variable and post hoc paired *t*-tests with Bonferroni correction. Since an individual participant never responded within 100 to 150 ms, the missing value was complemented by mean *P*_200_ obtained from the others within the interval. We conducted a two-way (2 [gain-decrease and probability-decrease condition] * 30 [time intervals until gain/probability becomes 0]) repeated-measures ANOVA on mean response time across four repetitions. We conducted a Friedman test to compare the estimated weighting of simple reaction pattern in the gain-decrease condition ${\varpi }_{s\_gain}$, probability-decrease condition ${\varpi }_{s\_prob}$, and optimal weighting ratio ${\varpi }_{s\_opt}$. Post hoc Wilcoxon signed-rank test with Bonferroni correction was performed. *p* < 0.05 was considered statistically significant. Cohen’s *dz* for paired t-test, A partial *η*^2^ for ANOVA, Kendall’s *W* for Friedman test, and *Z* for Wilcoxon signed-rank test were used to report effect sizes.

## Supplementary information

Supplementary Information

## Data Availability

The data supporting the findings of this study are available from the corresponding authors upon request.
