# Supplementary material for: Risk aversion in the adjustment of speed-accuracy tradeoff depending on time constraints
Source: Sci Rep. 2019 Aug 13;9:11732. doi: 10.1038/s41598-019-48052-0 (PMC6692412; doi:10.1038/s41598-019-48052-0)
Supplement: Supplementary file 1 — Supplementary Information [file 41598_2019_48052_MOESM1_ESM.docx]

**Supplementary Information**

**Risk aversion in the adjustment of speed-accuracy tradeoff depending on time constraints**

Ryoji Onagawa, Masahiro Shinya, Keiji Ota, Kazutoshi Kudo

**Supplementary Figures**

**Supplementary Figure 1.** Histogram of response times for selecting Option 100 (gray) and Option 200 (black). The probability of selecting Option 200 ($P_{200}$) is plotted as a function of the response time binned in eight equal intervals (50 ms) from 100 to 500 ms. The histogram shows the distribution of the response time across equal intervals (50 ms). The black curve shows the model fit using modified logistic regression and indicates the relationship between the response time and $P_{200}$ (i.e., participant’s own speed-accuracy tradeoff). We estimated participant’s own SAT by fitting using a generalized linear model fit with modified Probit link function to the mean response time binned in eight equal intervals within 100 to 550 ms and the corresponding $P_{200}$ (red dots in Fig. 2A). $P_{200}$ is described by following equations; $P_{200}(t)=0.5+(1-0.5)F(t|\mu_{p},\sigma_{p})$, where t was time. $F(t|\mu_{p},\sigma_{p})$was the cumulative normal function. $\mu_{p}$ was a mean, and $\sigma_{p}$ was an own standard deviation for the cumulative normal function. $P_{200}\left( t \right)$ satisfy the constraint that $P_{200}\left( t \right)=1-P_{100}\left( t \right)$*.* These data showed that $P_{200}$ increased with longer response time. Since participant ID8 performed only the choice reaction, this participant was excluded from further analyses.

**Supplementary Figure 2.** Time change pattern of the expected outcomes. Each panel shows the individual data. The purple line shows the time change of the expected outcomes based on data from an individual participant. The depth of color indicated corresponding with the level of time constraint $\tau$. Time change patterns in each participant were almost the same among participants. This relationship between the response time and the expected outcomes was used to estimate the time change of the variance of possible outcomes in both conditions in each participant.

**Supplementary Figure 3.** Time change pattern of the variance of possible outcomes. Each panel shows the individual data. The blue line and red line show time change of the variance of possible outcomes in the gain-decrease condition and the probability-decrease condition, respectively. The depth of color indicated corresponding with the level of time constraint $\tau$. The difference in the time change of risk between conditions was almost the same among participants.

**Supplementary Figure 4.** Histograms of response times in each condition. Each panel shows that the response time histograms in each condition in each participant. The gray histograms indicate the frequency selecting Option 100, and the black histograms indicate the frequency selecting Option 200.

**Supplementary Figure 5.** Gaussian mixture model (GMM) fitting. The gray histogram indicates RT distribution of an individual participant (including all response data). The orange shaded area and green shaded areas show components of Gaussian distribution using GMM. The orange and green components indicate the simple reaction and choice reaction, respectively. These panels show that each participants response time distribution was bimodal consistently, indicating that almost all participants switched reaction pattern corresponding to the level of time constraints.

**Supplementary Figure 6.** Gaussian mixture model (GMM) fitting for each condition in each participant. Each panel shows the response time distribution in gain-decrease condition (A) or probability-decrease condition (B) in each participant. The orange and green shaded areas indicate the components of Gaussian distributions determined by the estimated weighting of the simple reaction in each condition.

**Supplementary Figure 7.** Estimation of optimal weighting of the simple reaction. The orange lines indicate the expected gain of simple reaction pattern corresponding to time constraint $\tau$. The glue lines indicate the expected gain of choice reaction pattern corresponding to$\tau$. Yellow lozenge shows the point that expected gain of simple reaction pattern is equivalent to that of choice reaction pattern, indicating the optimal strategy switch point.

**Supplementary Figure 8.** Validation of the number of Gaussian components in GMM fitting. We adopted GMM fitting with one to four components of Gaussian distribution to evaluate the response strategy of each participant. We used the small-sample corrected Akaike Information Criteria (AICc) for model comparison. AICc was improved by including two components but was not improved by adding more. Therefore, GMM fitting with two components of Gaussian distribution was sufficient to explain observed response data.

**Supplementary Table**

| ID | Speed-accuracy tradeoff (SAT) fitting | | Gaussian mixture model (GMM) fitting | | | | | | Weighting parameter in both conditions | |
| --- | --- | --- | --- | --- | --- | --- | --- | --- | --- | --- |
|  |  |  | **Simple reaction pattern** | | | **Choice reaction pattern** | | | **Gain.** | **Prob.** |
|  | $\boldsymbol{\mu}_{\boldsymbol{p}}$ | $\boldsymbol{\sigma}_{\boldsymbol{p}}$ | $\varpi_{s}$ | $\mu_{s}$ | $\sigma_{s}$ | $\varpi_{c}$ | $\mu_{c}$ | $\sigma_{c}$ | $\varpi_{s\_gain}$ | $\varpi_{s\_prob}$ |
| 1 | 245.11 | 59.61 | 0.20 | 165.22 | 18.90 | 0.80 | 338.17 | 63.06 | 0.10 | 0.37 |
| 2 | 303.37 | 38.75 | 0.23 | 159.50 | 10.58 | 0.77 | 288.31 | 69.08 | 0.08 | 0.53 |
| 3 | 321.97 | 30.71 | 0.12 | 167.51 | 21.12 | 0.88 | 337.64 | 62.23 | 0.18 | 0.14 |
| 4 | 305.66 | 23.22 | 0.29 | 177.37 | 19.24 | 0.71 | 383.78 | 64.46 | 0.16 | 0.36 |
| 5 | 300.36 | 35.56 | 0.24 | 187.98 | 26.51 | 0.76 | 374.09 | 61.16 | 0.11 | 0.21 |
| 6 | 245.57 | 27.73 | 0.12 | 156.64 | 16.96 | 0.88 | 298.71 | 50.92 | 0.03 | 0.23 |
| 7 | 289.39 | 20.79 | 0.18 | 154.90 | 19.27 | 0.82 | 333.29 | 46.87 | 0.12 | 0.18 |
| 8 | 267.57 | 8.67 | 0.61 | 309.91 | 27.98 | 0.39 | 358.62 | 64.98 | 0.60 | 0.62 |
| 9 | 346.091 | 45.79 | 0.38 | 168.56 | 17.07 | 0.62 | 357.37 | 82.64 | 0.26 | 0.56 |
| 10 | 348.41 | 51.46 | 0.11 | 208.50 | 15.94 | 0.89 | 394.54 | 70.04 | 0.00 | 0.34 |
| 11 | 331.61 | 25.11 | 0.49 | 176.38 | 25.82 | 0.51 | 388.01 | 63.92 | 0.28 | 1.00 |
| 12 | 272.04 | 55.02 | 0.40 | 155.09 | 15.24 | 0.60 | 319.93 | 82.43 | 0.00 | 0.79 |

**Supplementary Table 1.** Model fitting results. This table includes the results of SAT fitting, Gaussian Mixture Model fitting for all response data and the estimation of weighting parameter for each condition. $\mu_{p}$ and $\sigma_{p}$ were the parameters of a cumulative normal function. $\varpi$ is a weighting parameter. $\mu$ is a mean, and $\sigma$ is a standard deviation of Gaussian distribution in GMM fitting and the estimation of weighting parameter. Subscript *s* indicates the simple reaction, and Subscript *c* indicates the choice reaction.
